# CSPG4 expression in soft tissue sarcomas is associated with poor prognosis and low cytotoxic immune response

**DOI:** 10.1186/s12967-022-03679-y

**Published:** 2022-10-11

**Authors:** Laurys Boudin, A de Nonneville, Pascal Finetti, Léna Mescam, A Le Cesne, Antoine Italiano, Jean-Yves Blay, Daniel Birnbaum, Emilie Mamessier, François Bertucci

**Affiliations:** 1 Laboratory of Predictive Oncology, Centre de Recherche en Cancérologie de Marseille, Institut Paoli-Calmettes, Aix-Marseille Université, INSERM UMR1068, CNRS UMR725, Marseille, France; 2 Department of Medical Oncology, Institut Paoli-Calmettes, Marseille, France; 3 French Sarcoma Group, Lyon, France; 4 Department of Pathology, Institut Paoli-Calmettes, 232 Bd. Sainte-Marguerite, 13009 Marseille, France; 5 Department of Medical Oncology, Gustave Roussy, Villejuif, France; 6 Department of Medical Oncology, Institut Bergonie, Bordeaux, France; 7 Department of Medical Oncology, Centre Léon Bérard, UNICANCER &, Université Claude Bernard Lyon I, Lyon, France

**Keywords:** Soft tissue sarcomas, CSPG4, Immune response, Immune therapy

## Abstract

**Background:**

Soft tissue sarcomas (STS) are heterogeneous and pro-metastatic tumors. Identification of accurate prognostic factors and novel therapeutic targets are crucial. CSPG4 is a cell surface proteoglycan with oncogenic functions. It recently emerged as a potential target for immunotherapy, including cell therapy based on CSPG4-specific chimeric antigen receptor (CAR)-redirected cytokine-induced killer lymphocytes (CSPG4-CAR.CIKs) in STS. However, expression of CSPG4 is poorly known in STS so far.

**Methods:**

We analyzed CSPG4 gene expression in 1378 localized STS clinical samples, and searched for correlations with clinicopathological data, including disease-free survival (DFS), and with tumor immune features.

**Results:**

CSPG4 expression was heterogeneous across samples. High expression was associated with younger patients’ age, more frequent undifferentiated pleomorphic sarcoma and myxofibrosarcoma pathological subtypes, more frequent internal trunk tumor site, and more CINSARC high-risk samples. No correlation existed with pathological tumor size and grade, and tumor depth. Patients with high CSPG4 expression displayed 49% (95% CI 42–57) 5-year DFS *versus* 61% (95% CI 56–68) in patients with low expression (p = 3.17E−03), representing a 49% increased risk of event in the “*CSPG4*-high” group (HR = 1.49, 95% CI 1.14–1.94). This unfavorable prognostic value persisted in multivariate analysis, independently from other variables. There were significant differences in immune variables between “*CSPG4*-high” and “*CSPG4*-low” tumors. The "*CSPG4*-low" tumors displayed profiles suggesting higher anti-tumor cytotoxic immune response and higher potential vulnerability to immune checkpoint inhibitors (ICI). By contrast, the "*CSPG4*-high" tumors displayed profiles implying an immune-excluded tumor microenvironment, potentially induced by hypoxia, resulting from an immature chaotic microvasculature, and/or the presence of contractile myofibroblasts.

**Conclusions:**

Patients with “CSPG4-high” STS, theoretically candidate for CAR.CIKs, display shorter DFS and an immune environment unfavorable to vulnerability to CAR.CIKs, which could be improved by combining anti-angiogenic drugs able to normalize the tumor vasculature. By contrast, “CSPG4-low” STS are better candidates for immune therapy involving ICI.

**Supplementary Information:**

The online version contains supplementary material available at 10.1186/s12967-022-03679-y.

## Background

Soft-tissue sarcomas (STS) are rare and serious tumors of mesenchymal origin affecting children and adults [1]. These heterogeneous tumors encompass more than 100 distinct pathological subtypes associated with variable biological and clinical behaviors [2]. Despite the successful advances in surgical resection, radiotherapy, and chemotherapy, the outcome of patients with non-metastatic STS is still poor: the disease recurs in approximately 50% of patients, often with distant failure [3–6]. In the metastatic setting, the prognosis remains dismal with a 5-year survival rate inferior to 25% [7]. Currently, two issues represent major challenges in the management of STS. The first one is the improvement of prognostic factors that will help to better define the role, if any, of adjuvant chemotherapy. Historically associated with pathological grade [8] and size, and depth [9], the prognostic characterization is being refined, thanks to the contribution of genomic signatures such as CINSARC [10] or the immunologic constant of rejection (ICR) signature [11]. Clinical trials assessing prospectively the clinical utility of CINSARC (NCT03805022, NCT04307277) are ongoing. The second issue relies on the identification of novel therapeutic targets. The limited development of new chemotherapy drugs and targeted therapies during the last decade has not modified the overall prognosis, and doxorubicin remains the backbone of systemic treatment.

Chondroitin sulfate proteoglycan 4 (CSPG4), also called neural-glial 2 (NG2), is a cell surface proteoglycan, overexpressed in certain human cancers, with low expression in normal tissues and oncogenic roles in tumor growth and metastatic dissemination [12] *via* the promotion of cell proliferation, cell survival and drug resistance, angiogenesis, cell migration and invasion [13]. Inhibition of CSPG4 by gene deletion or treatment with anti-CSPG4 antibodies inhibits tumor growth in xenografts from some malignancies [14, 15]. Since CSPG4 is expressed in the mesenchymal progenitor cells [16, 17] and pericytes [18] from which STS are supposed to originate, its activation could play a role in sarcoma progression. Driving oncogenic mutations in *Ng2/Cspg4*-expressing cells leads to the formation of sarcomas [18]. During the last years, CSPG4 was described as a potential target of cellular immunotherapy in cancers [12]. CSPG4 is also known to influence activation, maturation, proliferation, and migration of different immune cell subsets suggesting likely interaction with immunotherapy efficiency [12].

Recent data suggested that the immune system might positively impact the outcome of patients with STS [11, 19, 20]. Several clinical trials testing immunotherapy based on immune checkpoint inhibitors (ICI) have been launched [21], but the results were relatively disappointing and remain controversial [22]. Identification of efficacy predictive markers, such as the presence of tertiary lymphoid structures [23, 24], is crucial in this so heterogeneous group of tumors. Another immunotherapy type is adoptive cellular therapy in which the T-cells are redirected by tumor antigen-specific chimeric antigen receptors (CAR-Ts). This approach, very effective in B-cell cancers [25–27], remains challenging in solid tumors [28]. One approach dedicated to improving the efficacy and safety of CAR-based therapies is the engineering of immune effectors different from αβT-lymphocytes, such as γδT-cells, natural killer (NK), NKT, or cytokine-induced killer (CIK) cells [29]. A recent study revealed the therapeutic potential of CSPG4-specific chimeric antigen receptor (CAR)-redirected cytokine-induced killer lymphocytes (CSPG4-CAR.CIKs) in STS [30]. In this study, the CSPG4-CAR.CIKs effectively targeted multiple STS pathological subtypes *in vitro* and *in vivo*. Antitumor activity against STS spheroids was associated with tumor recruitment, infiltration, and matrix penetration. *In vivo*, the CSPG4-CAR.CIKs delayed or reversed the tumor growth in three STS xenograft models (leiomyosarcoma, undifferentiated pleomorphic sarcoma, and fibrosarcoma).

Expression of CSPG4 is poorly known in STS. To our knowledge, only three studies [30–32] in the literature analyzed its expression in clinical samples, including respectively 251, 55, and 108 cases, but only the two smallest ones searched for correlations with tumor clinical features. To fill this gap and given the potential relevance of CSPG4 as a target for immunotherapy, we analyzed its expression in 1,378 localized STS clinical samples. We searched for correlations between expression and clinicopathological data, including disease-free survival (DFS), but also the components of the tumor immune landscape.

## Methods

### Patients and tumor samples

We collected clinicopathological and gene expression data of clinical STS samples from 15 public data sets through the National Center for Biotechnology Information (NCBI)/Genbank GEO, ArrayExpress databases, and authors' websites (Additional file 1: Table S1). The gene expression profiles had been generated using DNA microarrays or RNA-sequencing. The data sets were selected if the clinical and expression data, including *CSPG4* expression level, were available. The final data set included 1,378 clinical samples. The study was approved by our institutional board. We also analyzed cancer cell line data from the Dependency Map (DepMap) portal (https://depmap.org/portal; accessed on 05 April 2022) to compare the RPPA-based protein expression levels *versus* the RNA-seq-based mRNA expression levels of CSPG4.

### Gene expression data analysis

The pre-analytic processing of data was done as previously described [11]. Briefly, a first step of normalization was applied to each data set separately, by using quantile normalization for the already processed non-Affymetrix data, and Robust Multichip Average (RMA) with non-parametric quantile algorithm for the raw Affymetrix data. Normalization was done in R using Bioconductor and associated packages. We then mapped hybridization probes and kept the most variant one in a given data set when multiple probes mapped to the same GeneID. The already normalized TCGA RNAseq data were log_2_-transformed. Finally, we corrected the batch effects across the 15 studies by using z-score normalization, in which mean and standard deviation were measured on leiomyosarcoma samples. CSPG4 expression was analyzed as discrete variable (high *versus* low) by using its median expression level of the whole series as cut-off.

Because CSPG4 is a potential target of immunotherapy, we searched for correlations between its tumor expression and different immune variables. We applied to each data set separately several immunity-, fibroblastic- or vasculature-related multigene classifiers/scores: the tumor-infiltrating lymphocyte (TIL) score [33], the 24 Bindea’s innate and adaptive immune cell subpopulations and two Bindea’s vessels signatures [34], two fibroblast subsets’ classifications [35, 36], hypoxia, acidosis and lactic acidosis Gatza’s scores [37], the Immunologic Constant of Rejection (ICR) classifier [38], and metagenes associated representative of T-cell-inflamed signature (TIS) [39], of tertiary lymphoid structures (TLS) signature [40], of cytolytic activity score [41], and the antigen processing machinery (APM) score [42]. We also applied the CINSARC signature, now recognized as the most relevant prognostic signature in STS [10].

To explore the biological pathways linked to *CSPG4* expression in STS, we applied a supervised analysis to the largest transcriptomics data set [10] including 131 “*CSPG4-high*” and 179 “*CSPG4-low*” tumors (learning set), and used the remaining tumors as independent validation set, including 558 “*CSPG4-high*” and 510 “*CSPG4-low*” tumors. In the learning set (N=310), we compared the expression profiles of 18,606 genes between “*CSPG4-high*” and “*CSPG4-low*” tumors using a moderated t-test with empirical Bayes statistic (limma R packages) and false discovery rate (FDR) correction. Significant genes were defined by the following thresholds: p<5%, q<10% and fold change (FC) superior to |1.5x|. Ontology analysis of the resulting gene list was based on GO biological processes of the Database for Annotation, Visualization and Integrated Discovery (DAVID; david.abcc.ncifcrf.gov/). We tested the robustness of the resulting gene list in the remaining data sets defined as validation set (N=1068) by computing the Pearson correlation distance between each sample and the *CSPG4-high* profile defined in the learning set as the average expression of each significant gene in the *CSPG4-high* class. Validation samples with negative correlation were classified as “*CSPG4-low*-like” and those with positive correlations were classified as “*CSPG4-high*-like”. Supervised analyses also searched for differentially altered genes between “*CSPG4-high*” and “*CPG4-low*” STS at the DNA level using the copy-number alteration (CNA), mutation and methylome public data available on TCGA portal (https://portal.gdc.cancer.gov). We compared the CNA profiles using the available “GISTIC thresholded by genes” TCGA data between “*CSPG4-high*” (N=123) and “*CSPG4-low*” (N=132) tumors across 24,776 genes using a Fisher’s exact test and FDR correction. Significant altered genes at “one copy gain”, “amplification”, “one copy loss” and “homozygous deletion” were defined by the following thresholds: p<5% and q<10%. Next, we compared the mutation profiles between “*CSPG4-high*” (N=119) and “*CSPG4-low*” (N=126) tumors across 7492 filtered (genes mutated in at least one sample) using a Fisher’s exact test and false discovery rate (FDR) correction. Silent mutations were excluded from analysis. Significant mutated genes were defined by the following thresholds: p<5% and q<10%. Finally, we compared the methylation profiles between “*CSPG4-high*” (N=101) and “*CSPG4-low*” (N=118) tumors across 450,000 probes using a Student t-test and FDR correction. Significant methylation sites were defined by the following thresholds: p<5%, q<10% and fold change (FC) superior to |1.25x|.

### Statistical analysis

The continuous variables were described using median and range, and the discrete values using number and percentage. The correlations between CSPG4 expression-based groups and clinicopathological variables and molecular signatures were measured using the Fisher’s exact test or Student’s t-test when appropriate. The endpoint of prognostic analysis was the disease-free survival (DFS), calculated from the date of diagnosis until the date of distant relapse or death from any cause, whichever occurred first. The follow-up was measured from the date of diagnosis to the date of last news for event-free patients. Survivals were estimated using the Kaplan-Meier method and curves compared with the log-rank test. Uni- and multivariate prognostic analyses were done using Cox regression analysis (Wald test). The variables tested in univariate analysis were patients’ age and gender, pathological tumor type (liposarcomas (LPS), leiomyosarcomas (LMS), undifferentiated pleomorphic sarcomas (UPS), myxofibrosarcomas (MFS), others), grade (3, 1-2), and size, tumor depth (superficial, deep) and site (extremities, head and neck, internal trunk, superficial trunk), CINSARC-based risk (high, low) and the CSPG4-based classification (high, low). Multivariate analysis incorporated all variables with a p-value inferior to 5% in univariate analysis. The correlations of molecular immune/stromal variables with “CSPG4-high” *versus* “CSPG4-low” status of samples were assessed by logistic regression analysis with the glm function (R statistical package; significance estimated by specifying a binomial family for models with a logit link). All statistical tests were two-sided, and the significance threshold was 5%. Analyses were done with the survival package (version 2.43) from R software (version 3.5.2).

## Results

### Patients’ characteristics and CSPG4 expression

A total of 1378 localized STS samples were available for analysis. Their characteristics are summarized in Table 1. The median patients’ age was 61 years (range, 2–92), and 51% of cases were male. The pathological subtypes were represented by 476 (35%) liposarcomas (LPS), 311 (23%) leiomyosarcomas (LMS), 288 (21%) undifferentiated pleomorphic sarcomas (UPS), 100 (7%) myxofibrosarcomas (MFS), and 177 (13%) other subtypes. Fifty-one percent were grade 3. The median pathological tumor size was 10 cm (range 1.2-39.5), and the most frequent tumor sites were extremities in 42% of cases and internal trunk in 39% of cases. The tumor was deep-seated (under fascia) in 89% of cases. Sixty-two percent (62%) of samples showed a complex genetic profile and 53% a low-risk according to the CINSARC signature.

**Table 1:** Clinicopathological characteristics of patients and samples

Characteristics	N	All	*CSPG4 classes*		p-value*
			low	high	
Patient's age, median					
Years (range)	528	61 (2–92)	61 (10–92)	57 (2–90)	**0.032**
Gender					
Female	299	299 (49%)	151 (47%)	148 (52%)	0.316
Male	306	306 (51%)	168 (53%)	138 (48%)	
Tumor site					
Extremity	206	206 (42%)	124 (47%)	82 (36%)	**0.007**
Head and neck	9	9 (2%)	7 (3%)	2 (1%)	
Internal trunk	195	195 (39%)	87 (33%)	108 (47%)	
Superficial trunk	84	84 (17%)	46 (17%)	38 (17%)	
Tumor depth					
Deep	144	144 (89%)	73 (88%)	71 (91%)	0.706
Superficial	17	17 (11%)	10 (12%)	7 (9%)	
Pathological type					
Undifferentiated pleomorphic sarcoma	288	288 (21%)	163 (24%)	125 (18%)	**7.52E−21**
Leiomyosarcoma	311	311 (23%)	87 (13%)	224 (33%)	
Liposarcoma	476	476 (35%)	259 (38%)	217 (32%)	
Myxofibrosarcoma	100	100 (7%)	78 (12%)	22 (3%)	
Other	177	177 (13%)	86 (13%)	91 (13%)	
Pathological tumor size, median cm (range)	170	10.0 (1.2–39.5)	12.53 (1.6–36)	11.8 (1.2–39.5)	0.540
Pathological FNCLCC grade					
1–2	163	163 (49%)	88 (51%)	75 (48%)	0.693
3	166	166 (51%)	85 (49%)	81 (52%)	
Genetic profile					
Simple	508	508 (38%)	269 (40%)	239 (35%)	0.095
Complex	844	844 (62%)	406 (60%)	438 (65%)	
CINSARC risk					
Low-risk	724	724 (53%)	381 (55%)	343 (50%)	**0.046**
High-risk	654	654 (47%)	308 (45%)	346 (50%)	
TCGA SARC, iCluster					
1	48	48 (27%)	2 (2%)	46 (51%)	**6.51E−14**
2	27	27 (15%)	11 (12%)	16 (18%)	
3	55	55 (30%)	45 (50%)	10 (11%)	
4	9	9 (5%)	7 (8%)	2 (2%)	
5	42	42 (23%)	25 (28%)	17 (19%)	
Follow-up median, months (min–max)	610	28 (1–222)	32 (1–203)	24 (1–222)	0.624
DFS event	610	223 (27%)	119 (33%)	104 (43%)	**1.28E−02**
5-year DFS [95% CI]	610	56% (52–61)	61% (56–68)	49% (42–57)	**3.17E−03**

The significant p-values are in bold

*FNCLCC, Fédération Nationale des Centres de Lutte Contre le Cancer;* *, Student’s t-test for continuous variables and Fisher’s exact test for discrete variables

*CSPG4* mRNA expression varied among the 1,378 tumors with a range of intensities over four units in log2 scale (Additional file 2: Figure S1A), suggesting a heterogeneous expression across the samples. Using the available omics data of 343 cancer cell lines including four sarcoma cell lines, we showed an excellent correlation between the mRNA and protein expression levels of CSPG4, with a Spearman’s rank correlation coefficient (rho) mean equal to 0.85 (p=9E-102) (Additional file 2: Figure S1A).

### CSPG4 expression correlates with clinicopathological features

We searched for correlations between *CSPG4* expression, assessed as a discrete variable (high *versus* low) and clinicopathological features (Table 1). There was no significant correlation with patients’ gender, pathological tumor size, pathological grade, and tumor depth. A trend for correlation existed with the genetic profile (p=0.063), with more frequent complex profiles in “*CSPG4*-high” tumors (65% versus 60%, p=0.095). Significant correlations were found with patients’ age (p=3.21E−02), pathological subtype (p=7.52E−21), tumor site (p=7.11E−03), and the CINSARC class (p=4.59E−02). Briefly, we found younger median age in “*CSPG4*-high” tumors (57 versus 60 years), more UPS (24% versu*s* 18%) and MFS (12% versus 3%) in “CSPG4-low” tumors and more LMS (33% versus 13%) in “CSPG4-high” tumors, more frequent extremity site for “*CSPG4*-low” tumors (47% versus 36%) and internal trunk site for “*CSPG4*-high” tumors (47% versus 33%), and more CINSARC high-risk samples in “*CSPG4*-high” tumors (50% versus 45%).

### CSPG4 expression correlates with disease-free survival

**Fig. 1:**
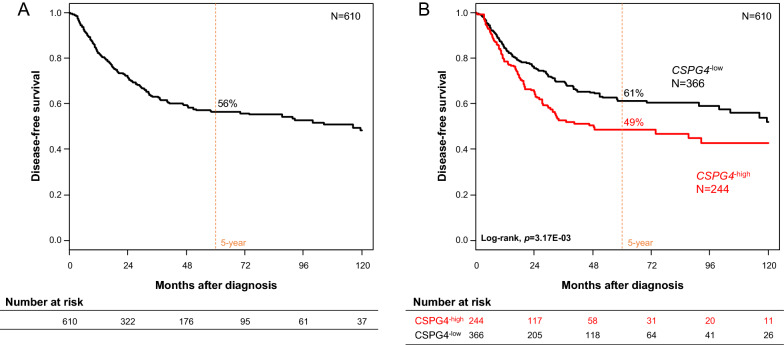
Disease-free survival in patients with localized STS after surgery. **A** Kaplan–Meier DFS curves in the 610 informative patients for DFS. **B** Similar to **A**, but according to CSPG4 expression (low and high). The p-value is for the log-rank test

The information on DFS and *CSPG4* expression was available for 610 patients. In the whole population, the median follow-up was 28 months (range 1-222), 223 DFS events occurred, and the 5-year DFS was 56% (95% CI 52–61) (Figure 1A). The 5-year DFS was 61% (95% CI 56–68) in the “CSPG4-low” subgroup and 49% (95%CI 42-57) in the “*CSPG4*-high” group (p=3.17E−03; Figure 1B). In univariate analysis for DFS (Table 2), this DFS difference corresponded to a 49% increased risk of event in the “*CSPG4*-high” group (HR=1.49, 95% CI 1.14–1.94; p=3.36E−03, Wald test). The other variables associated with shorter DFS included the pathological subtype (p=2.06E−05) and tumor size (HR=1.05, p=1.39E−03), and the CINSARC risk (p=4.35E−09). A trend was observed for the pathological grade (p= 0.098) and the tumor depth (p=0.062). No correlation was found between DFS and patients’ age and gender, and tumor site. In multivariate analysis, the “*CSPG4*-high” group (HR=3.47, 95% CI 1.73–6.95, p=4.59E−04) and higher pathological tumor size (HR=1.05, 95% CI 1.01–1.09, p=1.98E−02) remained significant, suggesting independent poor-prognosis value. The same result was observed when the ICR signature was added in the multivariate analysis (Additional file 7: Table S2).

**Table 2:** Uni- and multivariate prognostic analyses for DFS

Variables	Univariate			Multivariate		
	N	HR [95% CI]	p-value	N	HR [95% CI]	p-value
Patient's age	303	1.00 [0.99–1.01]	0.839			
Gender, male vs. female	303	1.04 [0.73–1.48]	0.831			
Tumor site, head and neck vs. extremity	382	0.00 [0.00- Inf]	0.118			
Tumor site, internal trunk vs. extremity		1.28 [0.90–1.81]				
Tumor site, superficial trunk vs. extremity		0.68 [0.40–1.16]				
Tumor depth, superficial vs. deep	128	0.26 [0.06–1.07]	0.062			
Pathological type, leiomyosarcoma vs. UPS	610	2.42 [1.65–3.54]	2.06E−05	55	3.63 [0.28–46.61]	0.322
Pathological type, liposarcoma vs. UPS		1.44 [1.00–2.06]		55	1.97 [0.10–37.16]	0.650
Pathological type, myxofibrosarcoma vs. UPS		1.01 [0.51–2.01]		55	7.02 [0.33–150.7]	0.213
Pathological type, other vs. UPS		0.78 [0.35–1.72]		55	0 [0.00—Inf]	0.999
Pathological tumor size (cm)	142	1.05 [1.02–1.09]	1.39E−03	55	1.07 [0.98–1.17]	0.155
Pathological FNCLCC grade, 3 vs. 1–2	239	1.47 [0.93–2.33]	0.098	55	6.84 [1.04–44.8]	0.045
Genetic profile, complex vs. simple	598	1.17 [0.89–1.54]	0.270			
CINSARC, high-risk vs. low-risk	610	2.22 [1.70–2.90]	4.35E−09	55	1.14 [0.26–4.93]	0,865
CSPG4, high vs. low	610	1.49 [1.14–1.94]	3.36E−03	55	10.47 [1.04–105.3]	**4.62E−02**

The significant p-values are in bold

*UPS* Undifferentiated Pleomorphic Sarcoma, *FNCLCC* Fédération Nationale des Centres de Lutte Contre le Cancer

### CSPG4 expression correlates with immune features

**Fig. 2:**
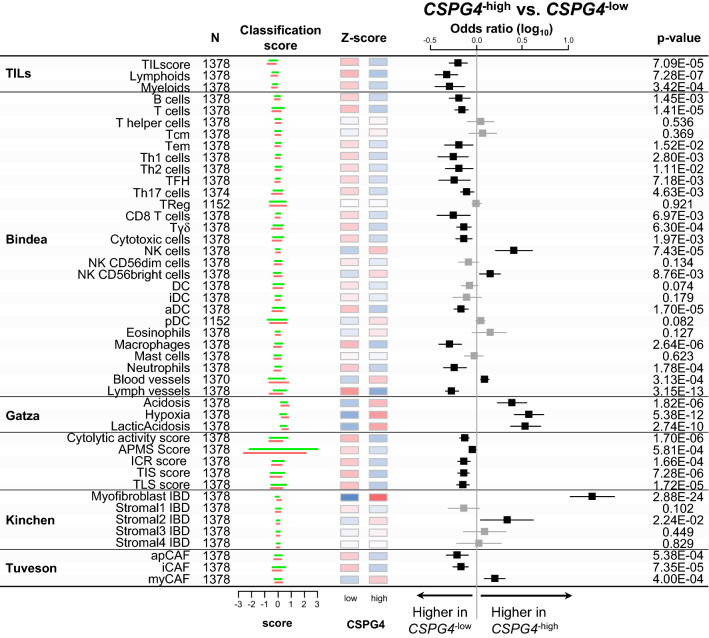
Correlations between CSPG4 expression and immune/stromal features. For each signature, the Z-score is displayed by color bars representing the 25 and 75 percentiles of “CSPG4-high” (red) and “CSPG4-low” (green). Average score of both CSPG4 classes are displayed by color coded boxes ranged from -3 (blue) to 3 (red) using white as mid color. Forest plots showing the Odds Ratios (log_10_) of immune and stromal signatures/scores in the “CSPG4-high” *versus* “CSPG4-low STS samples in univariate logistic regression analysis. The p-values are for the logit link tests: the black squares correspond to significant variables and the grey ones to non-significant variables

Next, we investigated whether *CSPG4* expression was associated with immunity-related parameters in STS samples (Figure 2). First, we compared the TIL scores in “*CSPG4*-high” and “*CSPG4*-low” tumors. These scores quantify the infiltration level of TIL, lymphoid and myeloid cells in tumor tissues, based on expression data. “*CSPG4*-low” STS displayed higher TILs scores (p=6.26E−05), notably with higher infiltration of lymphoid cells (p=5.11E−07) and myeloid cells (p=3.11E−04) than “*CSPG4*-high” STS, suggesting overall higher infiltration by immune cells in the former. Second, we looked at the composition and functional orientation of these tumor infiltrated cells, using immunity-related fibroblast-related and vasculature-related signatures/scores. Analysis of the 24 immune cell types defined as the immunome [27], fibroblast subsets and two vessels signatures showed significant differences between “*CSPG4*-high” and “*CSPG4*-low” tumors. “*CSPG4*-high” tumors displayed a higher infiltrate in NK cells (p=7.43E−05), notably the immunoregulatory ones (NK CD56^bright^ cells: p=8.76E−03). This was associated with a highly suppressive microenvironment, as demonstrated by the enrichment in signatures for acidosis (p=1.82E−06), lactic acidosis (p=2.74E−10), and hypoxia (p=5.38E−12) despite a higher signature of blood vessels (p=3.13E−04). *CSPG4*-high” tumors were also enriched in myofibroblasts CAFs (Kinchen_Myofibroblast: p=2.88E−24) and myCAFs (Tuveson_myCAF: p=4.00E−04) subsets. By contrast, the “*CSPG4*-low” tumors showed enrichment in signatures of macrophages (p=2.64E−06), T cells (p=1.41E−05), aDC cells (p=1.70E−05), neutrophils (p=1.78E−04), γδ T cells (p=6.30E−04), B cells (p=1.45E-03), cytotoxic cells (p=1.97E−03) and CD8 T cells (p=6.97E−03), higher inflammatory CAFs (Tuveson_iCAFs: p=7.35E−05) and antigen-presenting CAFs (Tuveson_apCAFs: p=5.38E−04), and higher lymph vessels signature (p=3.15E−13). Thus, except for NK cells, most immune cell types were observed in higher proportions in “*CSPG4*-low” tumors, which also had a less immune-suppressive environment. In parallel, analysis of other immune functional signatures confirmed these results. “CSPG4-high” samples showed a lower immune cytolytic activity score (p=1.70E−06) than “CSPG4-low samples”, as well as lower antigen processing/presentation machinery (APM) score (p=5.81E−04). They also displayed lower scores for signatures associated with response to ICI: Immunologic Constant of Rejection (ICR) score (p=1.66E−04), reflect of an antitumor cytotoxic immune response, T cell-inflamed signature (TIS) (p=7.28E−06), and tertiary lymphoid structure (TLS) score (p=1.72E−05).

### CSPG4 expression and correlations with biological variables

In order to further characterize the “*CSPG4-high*” and “*CGSPG4-low*” STS samples, we applied supervised analysis to transcriptomics, genomics and epigenomics data. Regarding transcriptomics data, the supervised analysis identified 379 genes differentially expressed between the “*CSPG4*-high” tumors and the “*CSPG4*-low” tumors of the learning set, including 229 genes upregulated and 150 genes downregulated in the “*CSPG4*-high” tumors (Additional file 8: Table S3, Additional file 3: Figure S2A). The robustness of this gene list was confirmed in the validation set including 1,068 tumors (Additional file 3: Figure S2B). Ontology analysis (Additional file 9: Table S4) revealed a net overrepresentation of immune ontologies among the genes overexpressed in the “*GSPG4-low*” tumors, whereas the genes overexpressed in the “*GSPG4-high*” tumors were associated with multiple ontologies such as those related to cell migration, cytoskeleton, collagen and extra-cellular matrix, response to stress, growth and development.

Using the TCGA data set [43], we then applied supervised analyses to identify the DNA copy number alterations (CNA), the gene mutations and the gene methylations differentially observed between the two sample groups. Regarding the comparison of CNA between the “*CSPG4-high*” and “*CGSPG4-low*” tumors, the profiles were very similar; no significant difference was observed regarding “one copy gains”, “amplifications”, and “homozygous deletions“, whereas 2255 genes were differentially altered regarding the frequency of “one copy loss”, including 255 genes more frequently lost in the ”*CSPG4-high*” samples and 2292 genes more frequently lost in the ”*CSPG4-low*” samples (Additional file 10: Table S5, Additional file 4: Figure S3). The comparison of mutational profiles showed no significant difference in term of mutation frequency between the “*CSPG4-high*” and “*CGSPG4-low*” tumors (Additional file 11: Table S6, Additional file 5: Figure S4). Three genes were more frequently mutated in the “*CSPG4-high*” samples (*RB1, PTEN, PRKDC*) and two were more frequently mutated in the “*CSPG4-low*” samples (*PEG2, AHK2*), but none of them remained significant after FDR correction. The comparison of methylation sites between the “*CSPG4-high*” and “*CGSPG4-low*” tumors identified 84 significant sites, including 35 more methylated in the “*CSPG4-high*” group and 49 in the “*CGSPG4-low*” group (Additional file 12: Table S7, Additional file 6: Figure S5).

Finally, we searched for correlation between the CSPG4 expression status and the five multi-omics subtypes defined in the TCGA set by iClustering integrating DNA copy number, DNA methylation, and expression of mRNA and miRNA [43]. As shown in Table 1, the correlation was highly significant : the “CSPG4-high” tumors were more frequently classified as iClusters 1 and 2, whereas the “CSPG4-low” tumors were more frequently classified as iCluster 3, 4 and 5 (p=6.51E−14).

## Discussion

In this series of 1378 STS clinical samples, high *CSPG4* expression was an independent unfavorable prognostic factor for DFS and was associated with low cytotoxic immune response. To our knowledge, this is the largest study analyzing the expression of this new potential target for immune therapy in STS.

Our analysis was based on gene expression of *CSPG4* in a very large series of clinical samples. The strong correlation between mRNA and protein expression levels of CSPG4 that we evidenced in 343 cancer cell lines suggests that CSPG4 protein expression parallels observations made at the transcriptomic level. Such mRNA level analysis allowed not only to avoid the classical limitations of immunohistochemistry (availability of antibodies, standardization, positivity cut-off, interpretation subjectivity…), but also to work on a large series of clinical samples and to search for correlations with expression of biologically and clinically relevant immune signatures. We found heterogeneous expression of *CSPG4* in clinical STS samples, as reported in the three previous studies on STS, all also performed at the transcriptional level [30–32]. Benassi *et al*. profiled 55 samples [31], Cattaruzza *et al.* 108 samples including the 55 previous ones [32], and Leuci *et al*. analyzed 251 TCGA samples [30]. Benassi *et al.* focused their analysis on 55 deeply localized, >5-cm diameter and high-grade lesions [31] and did not find any significant correlation between the *CSPG4* expression and clinicopathological variables. The same team [32] extended this series to a total of 108 cases and found higher expression in synovial sarcoma. Leuci *et al* did not search for eventual correlations [30]. In our present study, we found that expression was mainly associated with the pathological type, with higher expression in LMS and MFS and lower expression in UPS and LPS. Other significant correlations existed with age, tumor site, and CINSARC risk, the “*CSPG4*-high" samples being more frequently high-risk according to CINSARC than the “*CSPG4*-low” samples.

Our prognostic analysis included a large series of 610 patients informative for DFS, the largest prognostic study reported so far. In uni- and multivariate analyses, high *CSGP4* expression was associated with a higher risk of DFS event, independently from other prognostic variables including pathological tumor size and grade and CINSARC. The 5-year DFS was 61% in the “*CSPG4*-low” subgroup *versus* 49% in the “*CSPG4*-high” group, representing a 49% increased risk of event in the “*CSPG4*-high” group. These results are consistent with the sole other prognostic study published in the literature [31]. In a series of 108 patients with STS deeply localized, >5-cm diameter and grade 2–3 [32], the authors found higher *CSPG4* expression in the metastases when compared with paired primary lesions and when compared with normal lung and other tissues. They confirmed this result at the protein level using IHC based on a monoclonal antibody generated by their own team, with high expression on the surface of neoplastic cells and neovascular structures of primary and secondary tumor masses. Finally, they demonstrated in multivariate analysis the independent unfavorable prognostic value of high *CSPG4* expression for MFS [32]. This prognostic impact in STS was investigated at the functional level. Using *in vitro* and *in vivo* models, the same team [32] showed that CSPG4 controlled the tumor progression (local growth, cell adhesion, and motility, and cell survival) by mediating the interaction of sarcoma cells with the host extracellular matrix, in particular with collagen 6 (Col VI) that accumulates in the peri- and intra-lesional stroma. In another study [44], Hsu *et al*. showed that the effects of CSPG4 on STS growth depended of the tumor developmental stage: in established murine and human STS, inhibition of CSPG4, using anti-CSPG4 antibody immunotherapy or gene deletion, decreased the cell proliferation and tumor size and increased apoptosis, whereas Ng2/Cspg4 deletion at the time of tumor initiation resulted in the opposite effect on tumor growth. The prognostic value of *CSPG4* expression is likely tumor type-dependent. We recently showed the good-prognosis value of high *CSPG4* expression in a series of 309 GIST [45], whereas higher expression was associated with poorer prognosis in melanoma [46], glioblastoma [47], breast cancer [48], head and neck squamous cell carcinomas [49], and hepatocellular carcinoma [50].

The transmembrane proteoglycan CSPG4 had been originally identified by Dr Ferrone‘s team as a highly immunogenic tumor antigen on the surface of melanoma cells and was named *High Molecular Weight Melanoma-Associated Antigen* [51]. This antigen was then characterized by the same team in several cancers: melanoma [46], TNBC [14], malignant mesothelioma [15], acute myeloid leukemia [52], chordoma [53], glioblastoma [54], and osteosarcoma [55]. It is now identified as a potential therapeutic target for immune therapy in different cancers, including anti-idiotypic antibodies in melanoma [56–58], monoclonal antibodies in triple-negative breast cancer [14] and melanoma [59], antibody-drug conjugate in melanoma [60], and CAR-T cells in many cancers [61]. In sarcomas, Leuci *et al.* recently demonstrated *in vitro* and *in vivo* the anti-tumor activity of CSPG4-CAR.CIKs in STS pre-clinical samples. We thus searched for eventual correlations between *CSPG4* expression and immune/stromal features. Several of them were differentially enriched between "*CSPG4*-high" and "*CSPG4*-low" STS. "*CSPG4*-low" tumors had higher scores for immune signatures suggesting higher infiltration by immune cells, higher lymphatic vasculature and higher anti-tumor immune response. They were also associated with the presence of TLS and other signatures indicative of better response to ICI treatment [23], despite signs of exhaustion. In line with this, "*CSPG4*-low" tumors showed higher iCAFs and apCAFs, which respectively secrete cytokines to attract and entrap lymphocytes to turn them into harmless cells. Altogether, these data suggested that "*CSPG4*-low” STS could be better candidates for immune therapy involving ICI, which will fully unleash pre-infiltrated CD8 T cells cytotoxic potential [45]. By contrast, the "*CSPG4*-high" tumors displayed immune profiles suggesting an immune desert or immune-excluded tumor microenvironment, lacking all kinds of immune cells required to mount an effective anti-tumor response, except for the NK^bright^ cells. This lack of immune infiltrated cells can be related to the presence of myofibroblasts and myCAFs, which are fibroblastic subsets endowed with contractile features, which affect the distribution of blood vasculature [62]. They also secrete a massive amount of matrix and prevent lymphocyte accessibility to tumor cells. For all these reasons, immune desert tumor microenvironments have been reported to have limited sensitivity to ICI. In the study reported by Leuci *et al.*, the tumor elimination *in vitro* after treatment with CSPG4-CAR.CIKs was dependent on the expression level of tumor cells, suggesting that "*CSPG4*-high" STS should represent the most candidate population for such treatment [30]. But our present data suggest that a treatment based on CSPG4-CAR.CIKs infiltrating cells that will target "*CSPG4*-high" tumor cells could be interesting if some aspects responsible for the immune desert can be overcome first. Notably, immune desert can be induced by hypoxia. Hypoxia is a key determinant of tumor aggressiveness, therapy resistance and has a dampening effect on antitumor immune responses and immune cells recruitment. Hypoxia and lactic acidosis induce the functional suppression of NK cells. We found that both hypoxia and lactic acidosis were enhanced in "*CSPG4*-high" tumors. This observation is consistent with a certain overexpression of CSPG4 induced by the chronic hypoxia *in vitro* [63] and likely explains why only immunoregulatory NK^bright^ cells are present in "*CSPG4*-high" tumors. A strategy based on CSPG4-CAR.CIKs would thus require to overcome the functional suppression. Hypoxia results from an immature chaotic microvasculature within the tumor. Strategies that seek to normalize the tumor vasculature, such as tyrosine kinase inhibitors (TKIs) that target pro-angiogenic receptors, should help reduce hypoxia, enhance tumor’s perfusion and optimize therapy uptake. This would be a key point to improve the efficacy of CSPG4-CAR.CIKs in STS.

Supervised analysis of transcriptomics data between the “*CSPG4-high*” and “*CSPG4-low*” tumors confirmed the immune desert observed in the “*CSPG4-high*” tumors and their enrichment in genes related to cell migration, collagen and extra-cellular matrix, response to stress, growth and development. That might explain in part their poorer prognosis when compared with the “*CSPG4-low*” tumors. No significant difference was observed regarding the frequency of gene amplification or gene deletion between both tumor groups, notably for *CSPG4*, suggesting that the DNA copy number is not responsible for the *CSPG4* differential expression. Similarly, no gene showed a significant difference in term of mutation frequency between the “*CSPG4-high*” and “*CSPG4-low*” tumors. By contrast, 84 sites were differentially methylated between both tumor groups, calling for further investigations in order to assess an eventual functional link with CSPG4.

## Conclusion

To our knowledge, our study is the largest one describing CSPG4 expression in clinical cancer samples, here in STS. We show that expression of CSPG4 in STS samples is heterogeneous and associated independently with shorter DFS and with an immune landscape not favorable to anti-tumor cytotoxic response. The main strength of our study lies in the high number of rare tumor samples analysed. Others include its originality and analysis of correlations with tumor immune variables. Limitations include the retrospective nature and associated biases, analysis of bulk samples, and absence of analysis at the protein level. Obviously, analysis of larger clinical series and protein analysis are warranted. Yet, our results suggest the prognostic value of CSPG4 expression in STS and describe the immune microenvironment of tumors candidate to specific immune therapy such as CSPG4-CAR.CIKs. Whether patients with STS, and what type of patients, will benefit from such immunotherapy deserve rapid assessment in prospective clinical trials.

## Supplementary Information

**Additional file 1:** **Table S1.** (File format .xls). List of soft tissue sarcoma data sets included.

**Additional file 2:** **Figure S1.** (File format .ppt). CSPG4 expression in clinical STS samples and cancer cell lines. **A/** Box plot of mRNA expression levels in the 1,378 STS clinical samples. **B/** Spearman’s rank correlation coefficient (rho) between mRNA (RNAseq data) and protein (RPPA) expression in 343 cancer cell lines (grey) including four sarcoma cell lines (orange).

**Additional file 3: Figure S2.** (File format .ppt). Identification and validation of the CSPG4 gene expression signature in STS samples. **A/** Identification of the signature in the Chibon’s learning set (N=310). *Left,* Volcano-plot showing the 379 genes differentially expressed between “*CSPG4-high*” *versus* “*CSPG4-low*” STS samples. *Middle,* box plot of classification score (Pearson correlation, r) between both CSPG4 classes (Student t-test) associated with cross-table between observed and predicted CSPG4 groups (Fisher’s exact test)*.* **B/** Validation in the remaining public data sets (N=1,068). Box plot of classification score (Pearson correlation, r) between both CSPG4 classes (Student t-test) associated with cross-table between observed and predicted CSPG4 groups (Fisher’s exact test).

**Additional file 4: Figure S3.** (File format .ppt). Comparison of the CNA profiles between the “*CSPG4-high*” (N=123) and “*CSPG4-low*” (N=132) tumors. **A/** Heatmap of CNA in which the 24,776 genes were sorted by chromosomal location and the 255 STS samples were sorted by their *CSPG4* mRNA expression level. **B/** Left, frequency plots of CNA in both CSPG4 classes following four CNA levels: one-copy-gain (red), amplification (dark red), one-copy loss (green) and homozygous deletion (dark green). Loss alteration frequencies were negatively weighted. Right, *s*upervised analysis of CNA frequencies between “*CSPG4-high” vs. “CSPG4-low*” tumors. Plotted values represent the –log10 corrected p-values of the Fisher’s exact test weighted by the sign of the odds ratio for each CNA level. The vertical orange lines represents the significance thresholds (i.e. q<0.1).

**Additional file 5:** **Figure S4.** (File format .ppt). Distribution of mutations of the top 45 mutated genes in STS samples and comparison between the “*CSPG4-high*” (N=119) and “*CSPG4-low*” (N=126) tumors.** A/** Heatmap of mutations across 45 genes with mutation rate greater than 3% over all patients. Genes were sorted according their global mutation rate (blue bar plot to the right) and the 245 tumors were sorted by their *CSPG4* mRNA expression level. **B/** Bar plot of mutation frequency according to the “*CSPG4-high*” (red) or “*CSPG4-low*” (green) status. Orange plotted values represent the -log10 p-values of the Fisher’s exact test. The vertical dashed orange line represents the significance threshold (i.e. p<0.05), but no gene is significant after FDR correction (q-value).

**Additional file 6:** **Figure S5.** (File format .ppt). Comparison of methylation site profiles between the “*CSPG4-high*” (N=101) and “*CSPG4-low*” (N=118) tumors. **A/** Heatmap of methylation sites in which the 450,000 probes were sorted by chromosomal location and the 219 STS samples were sorted by their *CSPG4* expression level. **B/** Supervised analysis of methylation levels between the “*CSPG4-high” vs. “CSPG4-low*” tumors. The bar plotted values represent the –log10 corrected p-values of the Student t-test of the 84 significant probes with greater methylation in “CSPG4-high” (red) or in “CSPG4-low” (green).

**Additional file 7:** **Table S2.** (File format .xls). Uni- and multivariate prognostic analyses for DFS including the ICR signature.

**Additional file 8:** **Table S3.** (File format .xls). List of 379 genes differentially expressed between the “*CSPG4-high*” and “*CSPG4-low*” tumors.

**Additional file 9:** **Table S4.** (File format .xls). Ontologies associated with the 379 genes differentially expressed between the “*CSPG4-high*” and “*CSPG4-low*” tumors.

**Additional file 10:** **Table S5.** (File format .xls). Supervised analysis of CNA between the “*CSPG4-high*” (N=123) and “*CSPG4-low*” (N=132) tumors.

**Additional file 11:** **Table S6.** (File format .xls). Supervised analysis of mutation rates between the “*CSPG4-high*” (N=119) and “*CSPG4-low*” (N=126) tumors.

**Additional file 12:** **Table S7.** (File format .xls). List of the 84 sites differentially methylated between the “CSPG4-high” (N=101) and “CSPG4-low” (N=118) tumors.

** Additional file 13:** **Table S8.** (File format .xls). Expression data of each tumor normalised and used for the paper.

## Data Availability

All data generated or analysed during this study are included in this published article [and its supplementary information file: Additional file 13: Table S8.

## Abbreviations

STS: Soft tissue sarcomas
CSPG4: Chondroitin sulfate proteoglycan 4
NG2: Neural-glial
ICR: Immunologic constant of rejection
ICI: Immune checkpoint inhibitors
CAR: Chimeric antigen receptors
CSPG4-CAR.CIKs: CSPG4-specific chimeric antigen receptor (CAR)-redirected cytokine-induced killer lymphocytes
NCBI: National Center for Biotechnology Information
DFS: Disease-free survival
TIL: Tumor-infiltrating lymphocyte
TIS: T-cell-inflamed signature
TLS: Tertiary lymphoid structures
APM: Antigen processing machinery
LPS: Liposarcomas
LMS: Leiomyosarcomas (LMS)
UPS: Undifferentiated pleomorphic sarcomas
MFS: Myxofibrosarcomas
