# Supplementary material for: CSPG4 expression in soft tissue sarcomas is associated with poor prognosis and low cytotoxic immune response
Source: J Transl Med. 2022 Oct 11;20:464. doi: 10.1186/s12967-022-03679-y (PMC9552405; doi:10.1186/s12967-022-03679-y)
Supplement: Supplementary file 2 — Additional file 2: Figure S1. (File format .ppt). CSPG4 expression in clinical STS samples and cancer cell lines. A/ Box plot of mRNA expression levels in the 1,378 STS clinical samples. B/ Spearman’s rank correlation coefficient (rho) between mRNA (RNAseq data) and protein (RPPA) expression in 343 cancer cell lines (grey) including four sarcoma cell lines (orange). [file 12967_2022_3679_MOESM2_ESM.pptx]

## Slide 1
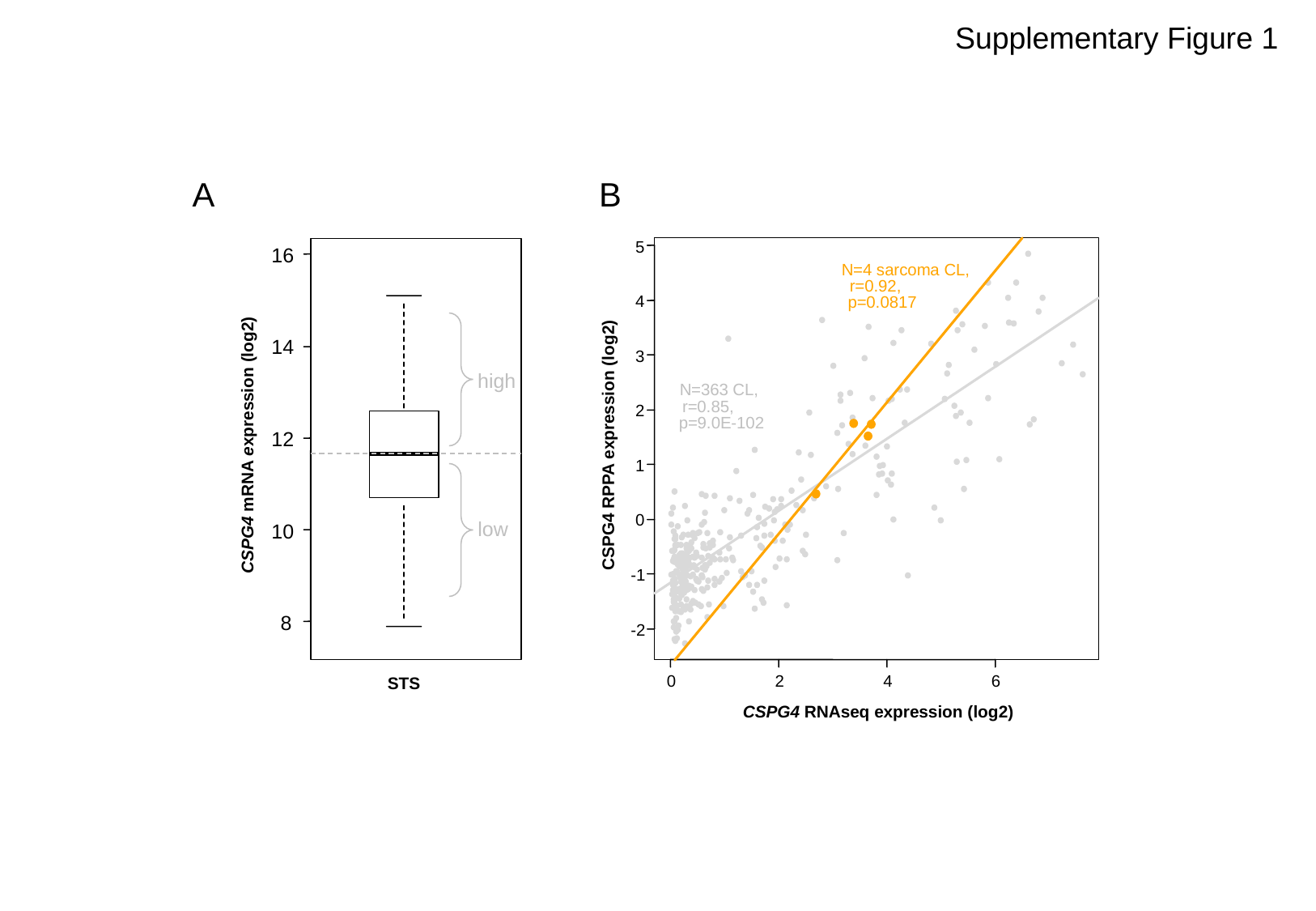

Supplementary Figure 1
A
B
5
N=4 sarcoma CL,
r=0.92,
p=0.0817
4
3
N=363 CL,
r=0.85,
p=9.0E-102
2
CSPG4 RPPA expression (log2)
1
0
-1
-2
0
2
4
6
CSPG4 RNAseq expression (log2)
8
STS
16
14
high
12
CSPG4 mRNA expression (log2)
low
10
