# Supplementary material for: CSPG4 expression in soft tissue sarcomas is associated with poor prognosis and low cytotoxic immune response
Source: J Transl Med. 2022 Oct 11;20:464. doi: 10.1186/s12967-022-03679-y (PMC9552405; doi:10.1186/s12967-022-03679-y)
Supplement: Supplementary file 3 — Additional file 3: Figure S2. (File format .ppt). Identification and validation of the CSPG4 gene expression signature in STS samples. A/ Identification of the signature in the Chibon’s learning set (N=310). Left, Volcano-plot showing the 379 genes differentially expressed between “CSPG4-high” versus “CSPG4-low” STS samples. Middle, box plot of classification score (Pearson correlation, r) between both CSPG4 classes (Student t-test) associated with cross-table between observed and predicted CSPG4 groups (Fisher’s exact test). B/ Validation in the remaining public data sets (N=1,068). Box plot of classification score (Pearson correlation, r) between both CSPG4 classes (Student t-test) associated with cross-table between observed and predicted CSPG4 groups (Fisher’s exact test). [file 12967_2022_3679_MOESM3_ESM.pptx]

## Slide 1
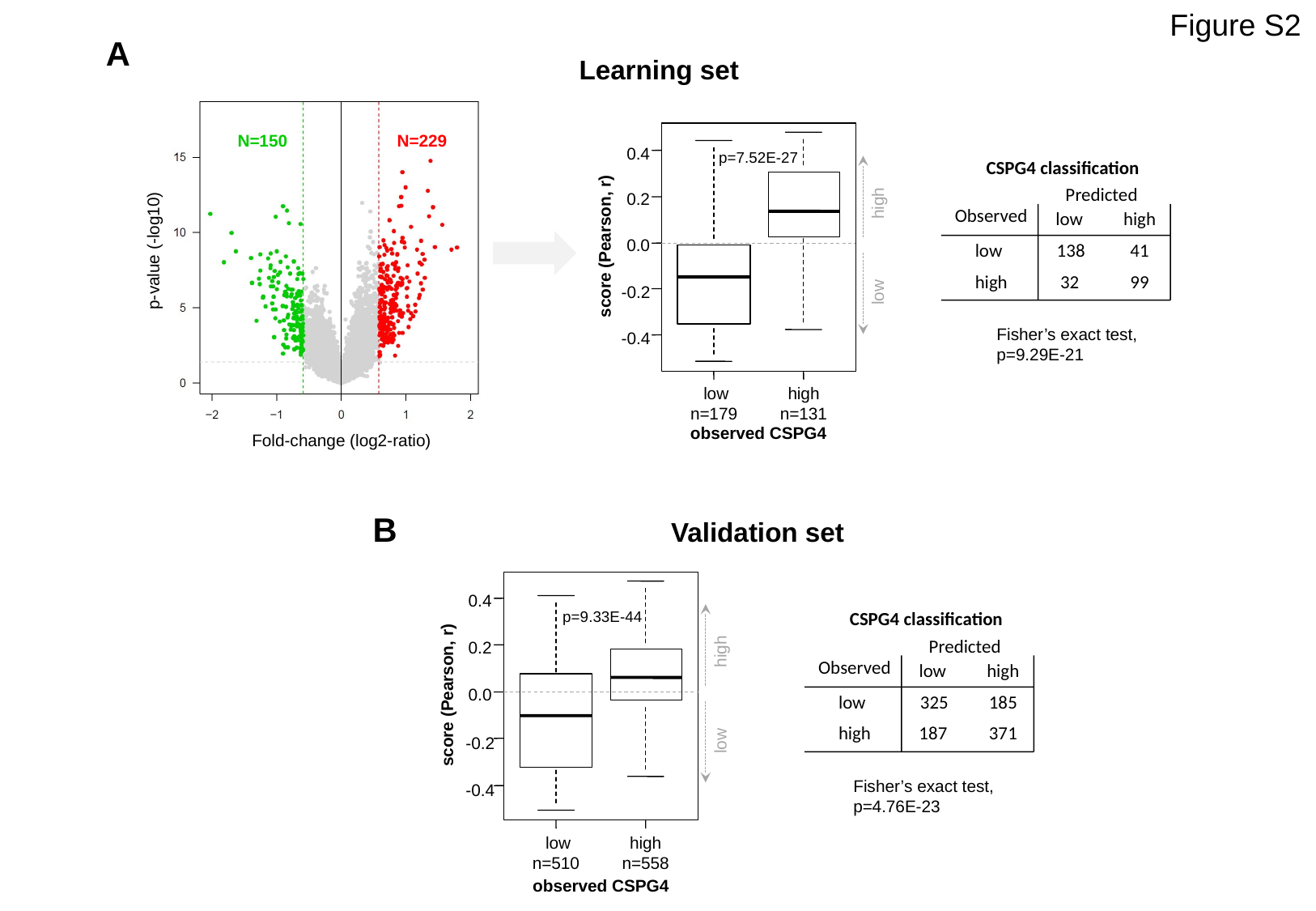

Figure S2
A
Learning set
N=150
N=229
p-value (-log10)
Fold-change (log2-ratio)
0.4
p=7.52E-27
0.2
0.0
score (Pearson, r)
-0.2
-0.4
 low
n=179
high
n=131
observed CSPG4
high
low
CSPG4 classification
Predicted
Observed
low
high
low
138
41
high
32
99
Fisher’s exact test,
p=9.29E-21
B
Validation set
0.4
p=9.33E-44
0.2
0.0
score (Pearson, r)
-0.2
-0.4
 low
n=510
high
n=558
observed CSPG4
high
low
CSPG4 classification
Predicted
Observed
low
high
low
325
185
high
187
371
Fisher’s exact test,
p=4.76E-23
