# Supplementary material for: CSPG4 expression in soft tissue sarcomas is associated with poor prognosis and low cytotoxic immune response
Source: J Transl Med. 2022 Oct 11;20:464. doi: 10.1186/s12967-022-03679-y (PMC9552405; doi:10.1186/s12967-022-03679-y)
Supplement: Supplementary file 4 — Additional file 4: Figure S3. (File format .ppt). Comparison of the CNA profiles between the “CSPG4-high” (N=123) and “CSPG4-low” (N=132) tumors. A/ Heatmap of CNA in which the 24,776 genes were sorted by chromosomal location and the 255 STS samples were sorted by their CSPG4 mRNA expression level. B/ Left, frequency plots of CNA in both CSPG4 classes following four CNA levels: one-copy-gain (red), amplification (dark red), one-copy loss (green) and homozygous deletion (dark green). Loss alteration frequencies were negatively weighted. Right, supervised analysis of CNA frequencies between “CSPG4-high” vs. “CSPG4-low” tumors. Plotted values represent the –log10 corrected p-values of the Fisher’s exact test weighted by the sign of the odds ratio for each CNA level. The vertical orange lines represents the significance thresholds (i.e. q<0.1). [file 12967_2022_3679_MOESM4_ESM.pptx]

## Slide 1
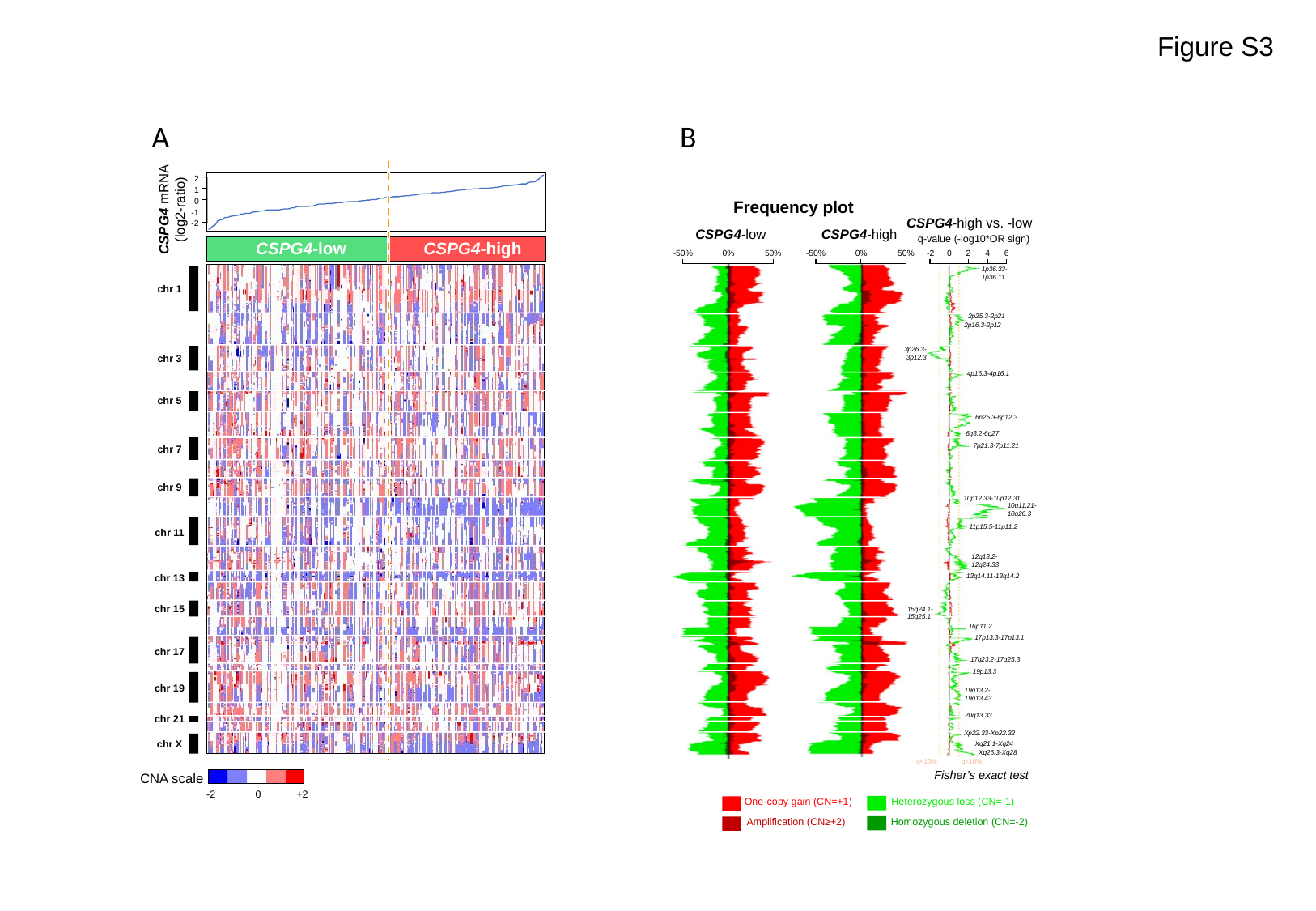

Figure S3
A
B
2
1
0
-1
-2
CSPG4 mRNA
(log2-ratio)
CSPG4-low
CSPG4-high
chr 1
chr 3
chr 5
chr 7
chr 9
chr 11
chr 13
chr 15
chr 17
chr 19
chr 21
chr X
CNA scale
-2
0
+2
Frequency plot
CSPG4-high vs. -low
q-value (-log10*OR sign)
-2
0
2
4
6
1p36.33-
1p36.11
3p26.3-
3p12.3
6p25.3-6p12.3
7p21.3-7p11.21
11p15.5-11p11.2
12q13.2-
12q24.33
13q14.11-13q14.2
16p11.2
17q23.2-17q25.3
Xq21.1-Xq24
Xq26.3-Xq28
Fisher’s exact test
q<10%
q<10%
CSPG4-low
-50%
0%
50%
CSPG4-high
-50%
0%
50%
19p13.3
19q13.2-
19q13.43
20q13.33
Xp22.33-Xp22.32
17p13.3-17p13.1
10p12.33-10p12.31
10q11.21-
10q26.3
4p16.3-4p16.1
2p25.3-2p21
2p16.3-2p12
15q24.1-
15q25.1
6q3.2-6q27
One-copy gain (CN=+1)
Amplification (CN≥+2)
Heterozygous loss (CN=-1)
Homozygous deletion (CN=-2)
