# Supplementary material for: CSPG4 expression in soft tissue sarcomas is associated with poor prognosis and low cytotoxic immune response
Source: J Transl Med. 2022 Oct 11;20:464. doi: 10.1186/s12967-022-03679-y (PMC9552405; doi:10.1186/s12967-022-03679-y)
Supplement: Supplementary file 5 — Additional file 5: Figure S4. (File format .ppt). Distribution of mutations of the top 45 mutated genes in STS samples and comparison between the “CSPG4-high” (N=119) and “CSPG4-low” (N=126) tumors. A/ Heatmap of mutations across 45 genes with mutation rate greater than 3% over all patients. Genes were sorted according their global mutation rate (blue bar plot to the right) and the 245 tumors were sorted by their CSPG4 mRNA expression level. B/ Bar plot of mutation frequency according to the “CSPG4-high” (red) or “CSPG4-low” (green) status. Orange plotted values represent the -log10 p-values of the Fisher’s exact test. The vertical dashed orange line represents the significance threshold (i.e. p<0.05), but no gene is significant after FDR correction (q-value). [file 12967_2022_3679_MOESM5_ESM.pptx]

## Slide 1
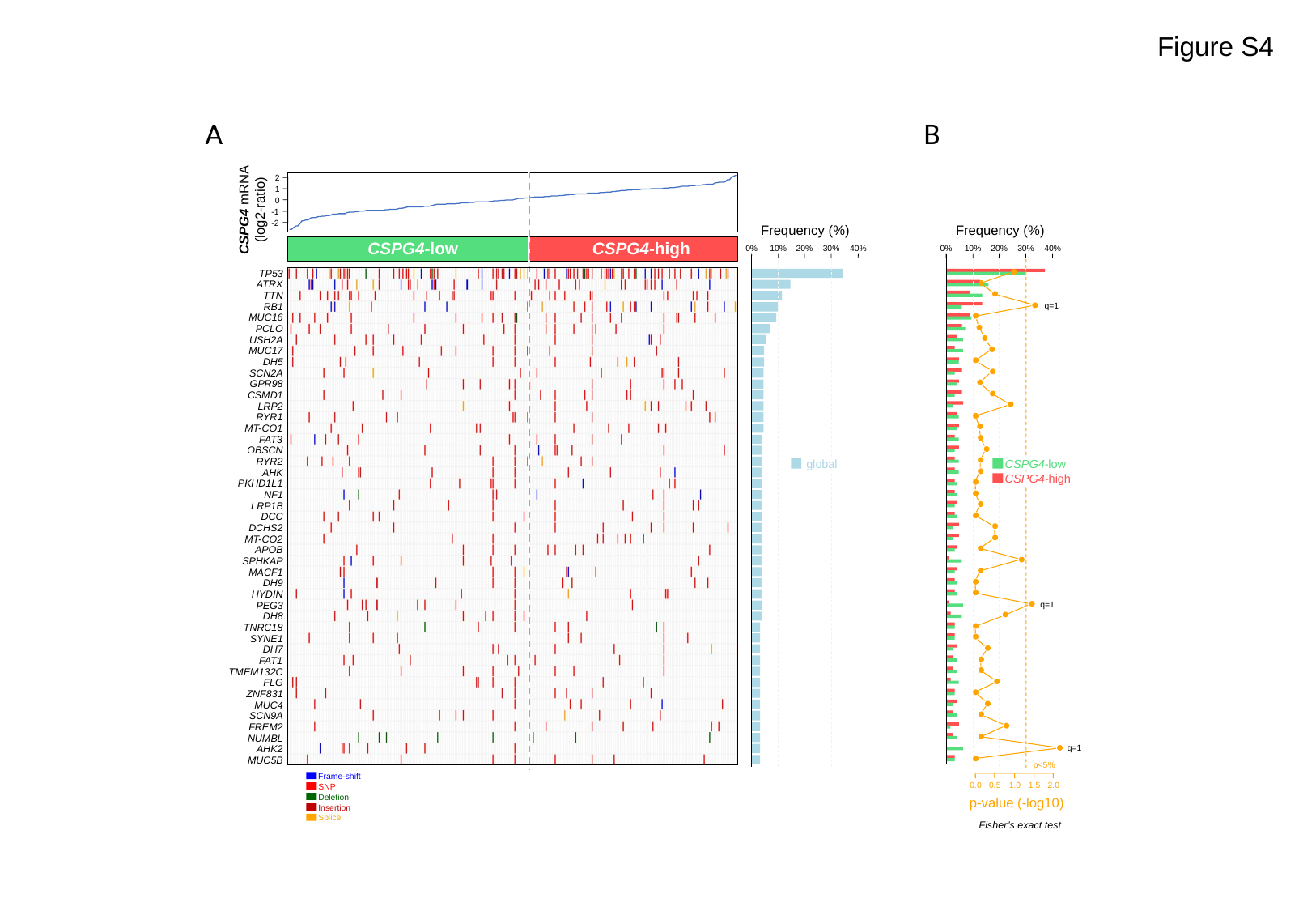

Figure S4
A
B
2
1
0
-1
-2
CSPG4 mRNA
(log2-ratio)
CSPG4-low
CSPG4-high
Frequency (%)
0%
10%
20%
30%
40%
TP53
ATRX
TTN
RB1
MUC16
PCLO
USH2A
MUC17
DH5
SCN2A
GPR98
CSMD1
LRP2
RYR1
MT-CO1
FAT3
OBSCN
RYR2
AHK
PKHD1L1
NF1
LRP1B
DCC
DCHS2
MT-CO2
APOB
SPHKAP
MACF1
DH9
HYDIN
PEG3
DH8
TNRC18
SYNE1
DH7
FAT1
TMEM132C
FLG
ZNF831
MUC4
SCN9A
FREM2
NUMBL
AHK2
MUC5B
global
Frame-shift
SNP
Deletion
Insertion
Splice
Frequency (%)
0%
10%
20%
30%
40%
q=1
q=1
q=1
p<5%
0.0
0.5
1.0
1.5
2.0
p-value (-log10)
CSPG4-low
CSPG4-high
Fisher’s exact test
