# Supplementary material for: CSPG4 expression in soft tissue sarcomas is associated with poor prognosis and low cytotoxic immune response
Source: J Transl Med. 2022 Oct 11;20:464. doi: 10.1186/s12967-022-03679-y (PMC9552405; doi:10.1186/s12967-022-03679-y)
Supplement: Supplementary file 6 — Additional file 6: Figure S5. (File format .ppt). Comparison of methylation site profiles between the “CSPG4-high” (N=101) and “CSPG4-low” (N=118) tumors. A/ Heatmap of methylation sites in which the 450,000 probes were sorted by chromosomal location and the 219 STS samples were sorted by their CSPG4 expression level. B/ Supervised analysis of methylation levels between the “CSPG4-high” vs. “CSPG4-low” tumors. The bar plotted values represent the –log10 corrected p-values of the Student t-test of the 84 significant probes with greater methylation in “CSPG4-high” (red) or in “CSPG4-low” (green). [file 12967_2022_3679_MOESM6_ESM.pptx]

## Slide 1
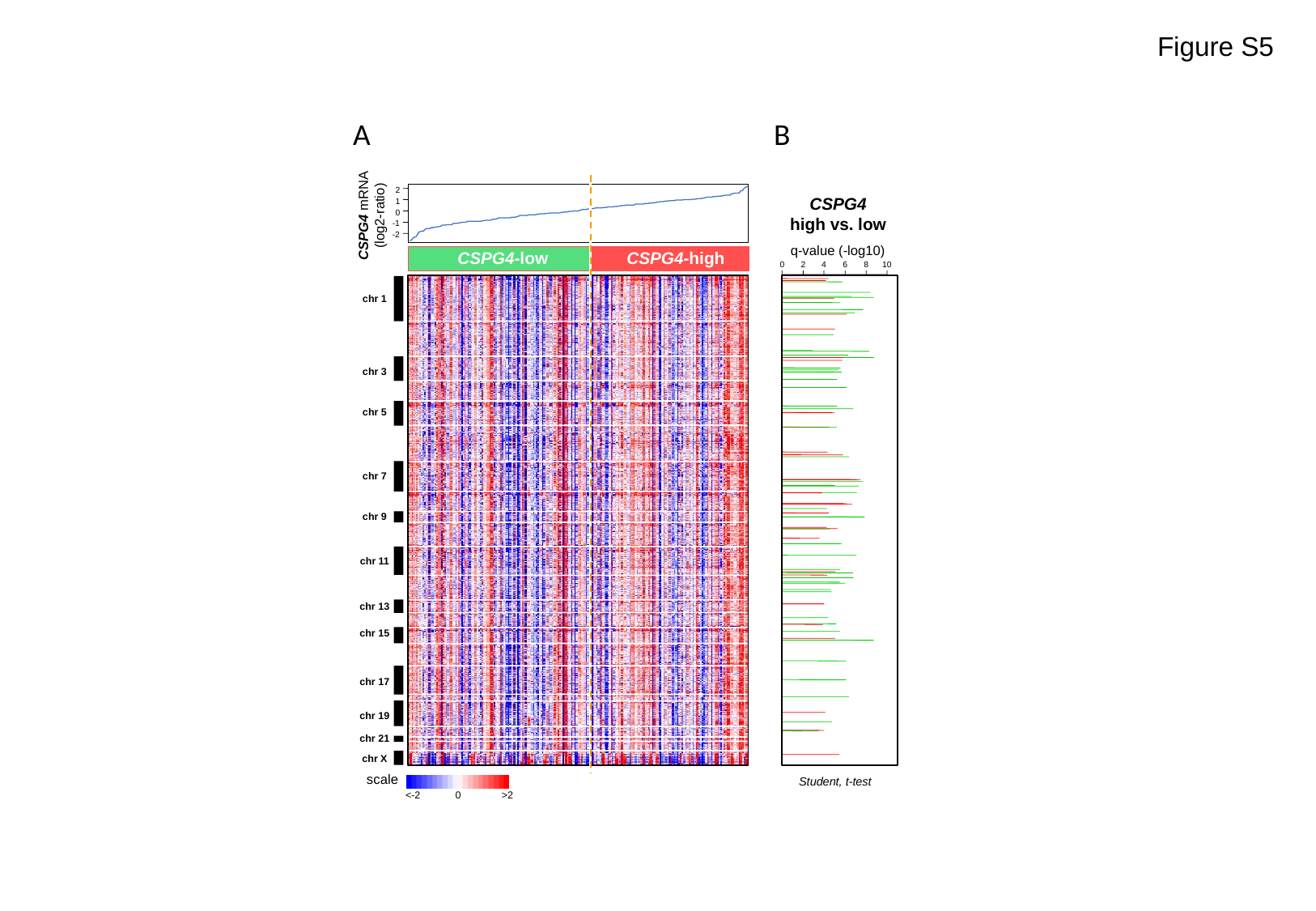

Figure S5
A
B
2
1
0
-1
-2
CSPG4 mRNA
(log2-ratio)
CSPG4-low
CSPG4-high
chr 1
chr 3
chr 5
chr 7
chr 9
chr 11
chr 13
chr 15
chr 17
chr 19
chr 21
chr X
scale
>2
<-2
0
CSPG4
high vs. low
q-value (-log10)
0
2
4
6
8
10
Student, t-test
